# Supplementary material for: Combination Antiretroviral Therapy and Immunophenotype of Feline Immunodeficiency Virus
Source: Viruses. 2023 Mar 24;15(4):822. doi: 10.3390/v15040822 (PMC10146003; doi:10.3390/v15040822)
Supplement: Supplementary file 1 [file viruses-15-00822-s001.zip › viruses-2267601-supplementary.pdf]

Supplementary Materials

Figure S1. Blood lineage markers in uninfected control and FIV+ cats.

Figure S2. Blood activation markers in uninfected control and FIV+ cats.

Figure S3. Lymph node lineage markers in uninfected control and FIV+ cats.

Figure S4. Lymph node activation markers in uninfected control and FIV+ cats.

Table S1. Complete statistical comparisons between uninfected control cats and FIV+ cats.

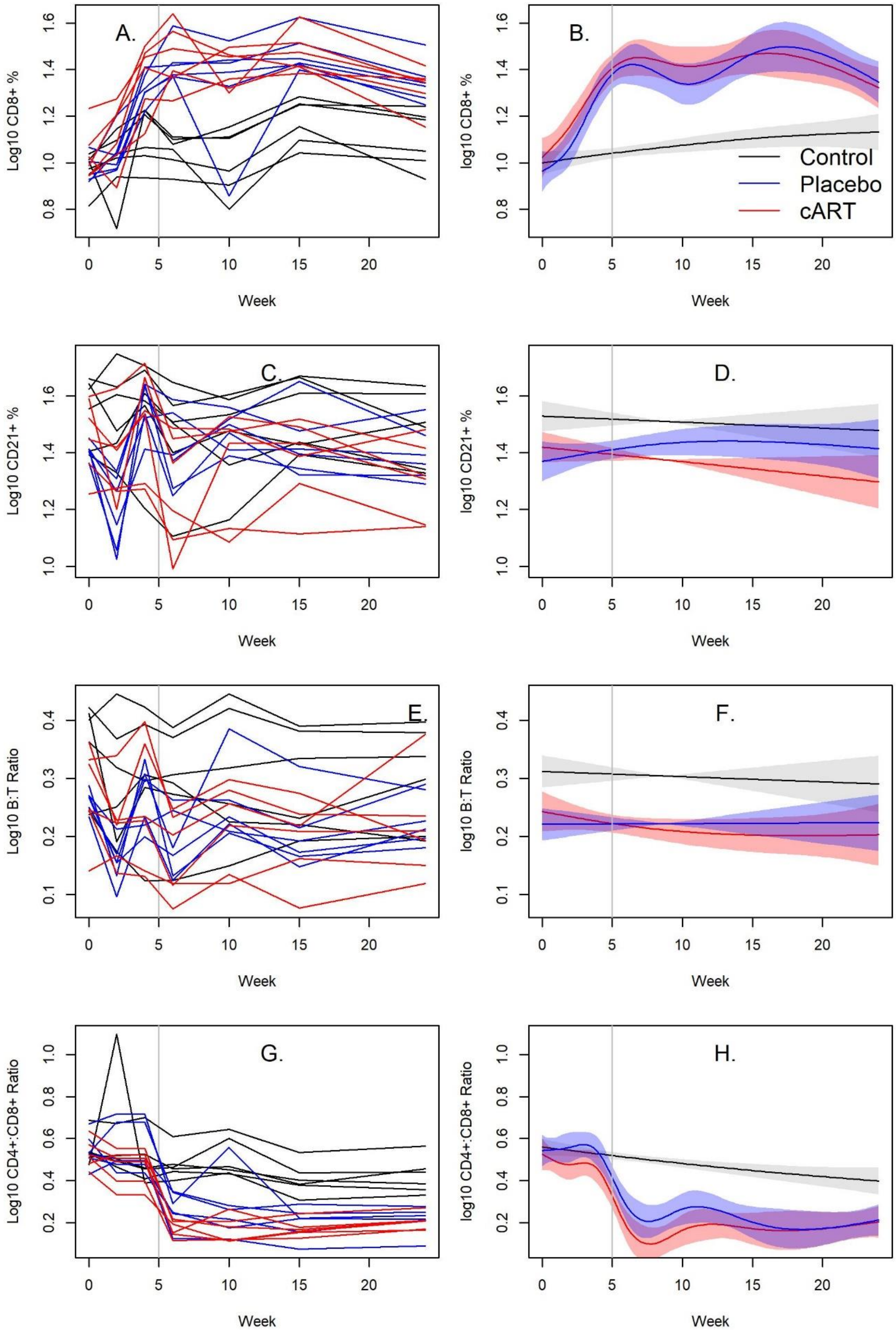

Figure S1. Blood lineage markers in uninfected control and FIV+ cats. There we no differences in the proportions of $CD8^+$, $CD21^+$, B:T, and $CD4^+$:$CD8^+$ T cells between cART and placebo-treated FIV+ cats. Uninfected control cats had lower ($p<0.01$) proportions of $CD8^+$ cells and higher ($p<0.01$) proportions of $CD4^+$:$CD8^+$ compared to all FIV+ cats. The vertical bar indicates when cART and placebo treatment began. (A, C, E, G) Line plots of individual cats over time, and (B, D, F, H) their associated spline fits from GAM analyses are presented.

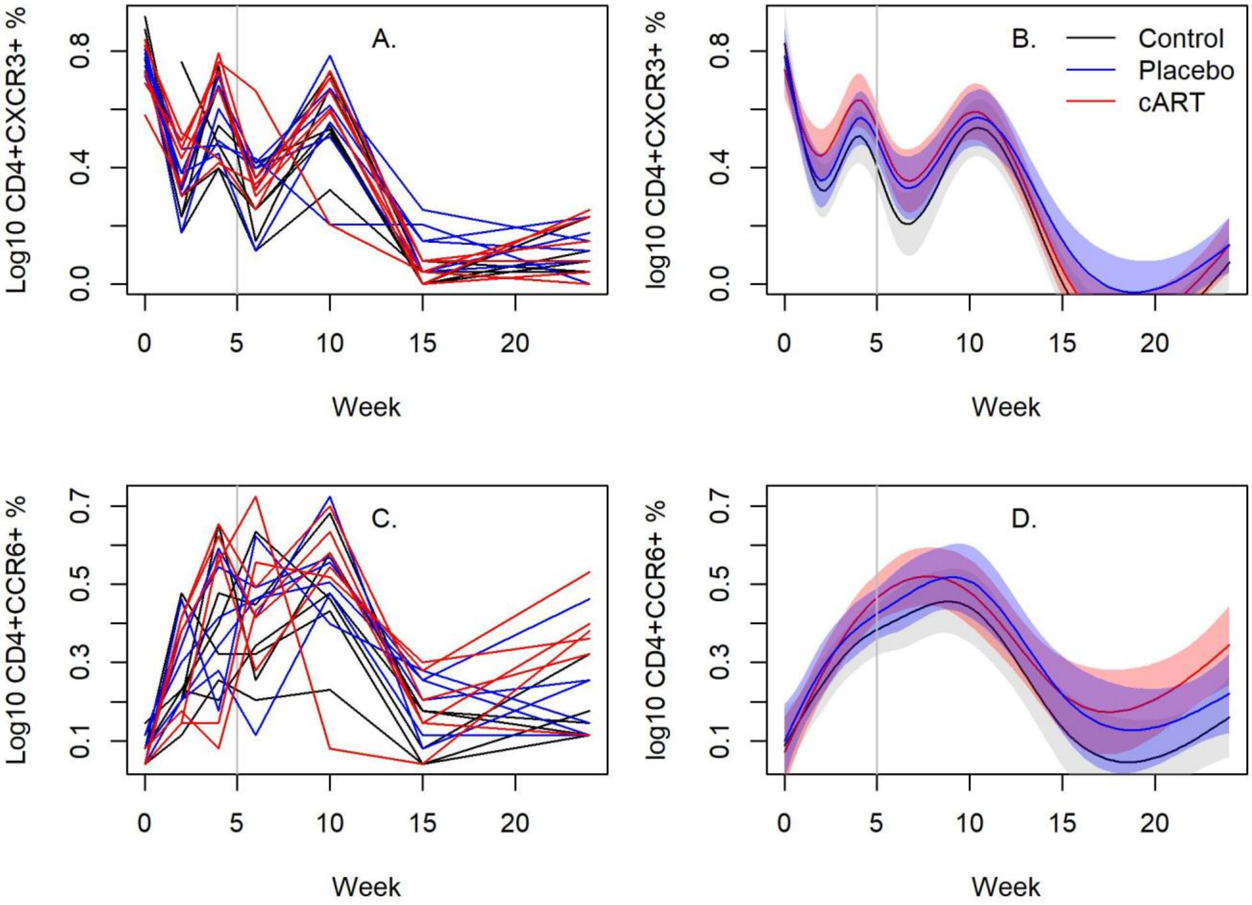

Figure S2. Blood activation markers in uninfected control and FIV+ cats. There were no differences in the proportions of $CD4^{+}CXCR3^{+}$ and $CD4^{+}CCR6^{+}$ cells between any treatment group across uninfected control, cART-treated FIV+, and placebo-treated FIV+ cats. The vertical bar indicates when cART and placebo treatment began. (A & C) Line plots of individual cats over time, and (B & D) their associated spline fits from GAM analyses are presented.

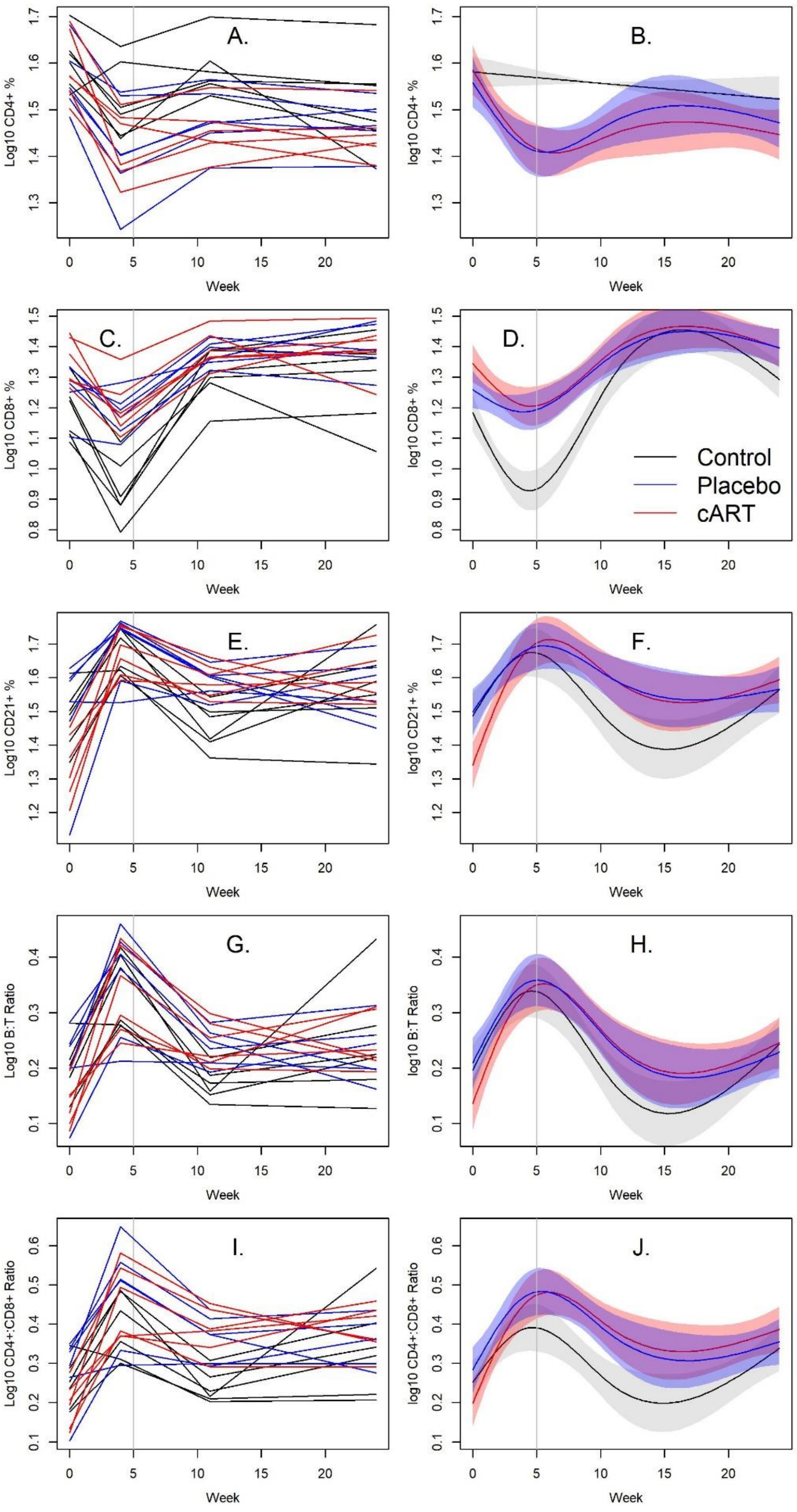

Figure S3. Lymph node lineage markers in uninfected control and FIV+ cats. There we no differences in the proportions of $CD4^+$, $CD8^+$, $CD4^+$:$CD8^+$, and B:T cells between cART and placebo-treated FIV+ cats. Proportions of $CD21^+$ T cells were different between all groups, but the lower values in the cART group prior to treatment caused the statistical difference between cART and placebo-treated FIV+ cats; their values were not different after treatment began. The vertical bar indicates when cART and placebo treatment began. (A, C, E, G, I) Line plots of individual cats over time, and (B, D, F, H, J) their associated spline fits from GAM analyses are presented.

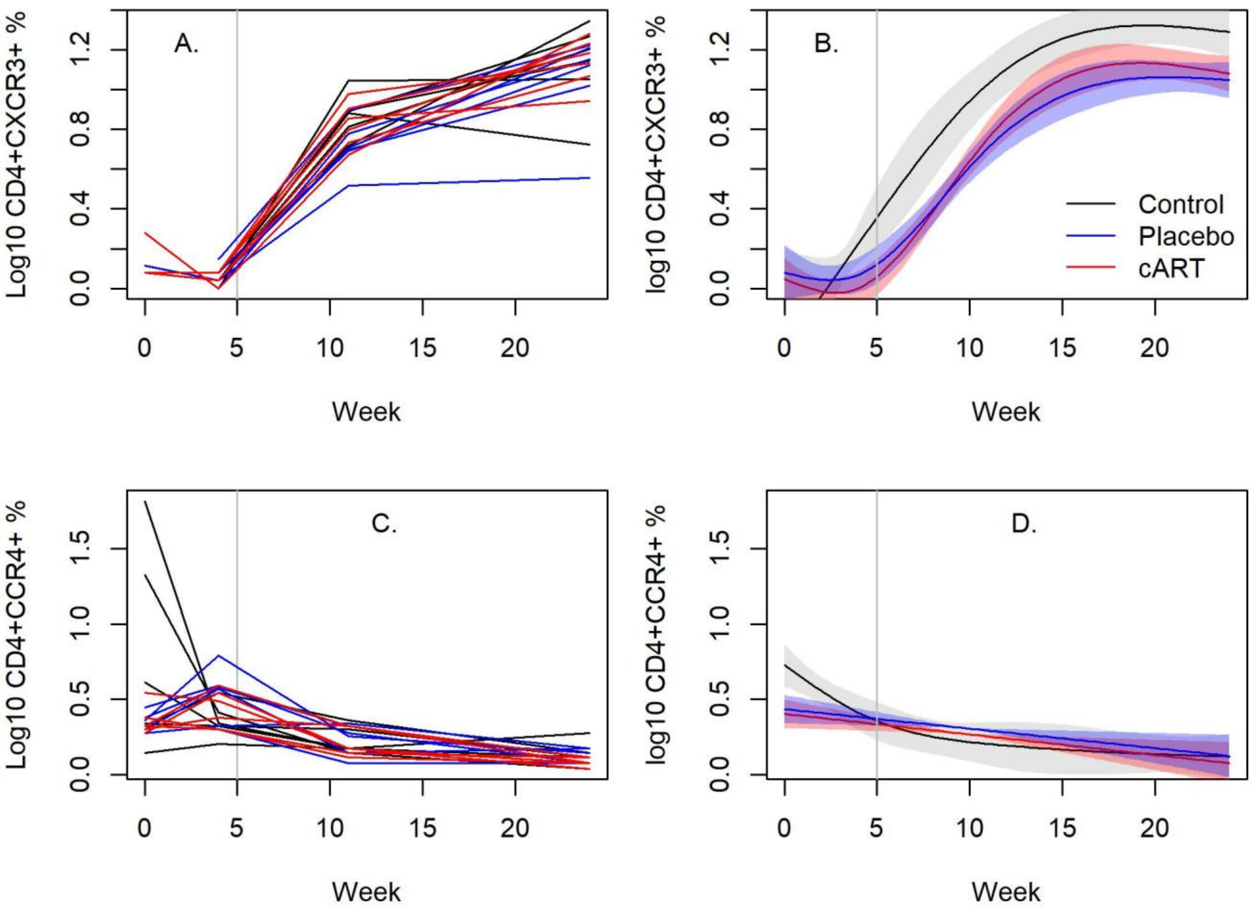

Figure S4. Lymph node activation markers in uninfected control and FIV+ cats. There were no differences in the proportions of $CD4^{+}CXCR3^{+}$ and $CD4^{+}CCR6^{+}$ cells between cART and placebo-treated FIV+ cats. The vertical bar indicates when cART and placebo treatment began. (A & C) Line plots of individual cats over time, and (B &D) their associated spline fits from GAM analyses are presented.

Table S1. Analyses of mean values across time of blood and saliva viral and proviral loads, hematology values, blood lineage and activation markers, and lymph node lineage and activation markers in uninfected control and FIV+ cats. The table shows comparisons between uninfected control, cART-treated FIV+, and placebo-treated FIV+ cats. The deviance demonstrates the fit of the model. Shared a, b, or c, values indicate lack of statistical significance, and differences in a, b, or c values highlight differences.

			Treatment	sWeek*Control	sWeek*Pacebo	sWeek*cART	Deviance (%)
Proviral and Viral Loads	Blood	F	1145	0	200	212	98.8
	Proviral load	P	<0.001	>0.999 a	<0.001 b	<0.001 b	
	Blood	F	816	0	85	78	96.5
	Viral load	P	<0.001	>0.999 a	<0.001 b	<0.001 b	
	Saliva	F	143	0	32	34	93.7
	Proviral load	P	<0.001	>0.999 a	<0.001 b	<0.001 b	
	Saliva	F	117	0	5	6	91.3
	Viral load	P	<0.001	>0.999 a	0.003 b	0.002 c	
Hematology	Neutrophil %	F	12	0.4	9	4	49.7
		P	<0.001	<0.001 a	<0.001 b	<0.001 c	
	Lymphocyte %	F	3	1	5	11	46.9
		P	0.055	0.320 a	<0.001 b	0.002 a	
Blood Lineage Markers	CD4%	F	5	5	48	18	72.6
		P	0.006	0.027 a	<0.001 b	<0.001 c	
	CD8%	F	17	4	27	22	85.7
		P	<0.001	0.002 a	<0.001 b	<0.001 b	
	CD21%	F	2	0.81	9	8	74.3
		P	0.144	0.329 a	<0.001 a	0.007 a	
	B:T ratio	F	3	0.43	0.01	3	75.5
		P	0.058	0.490 a	0.905 a	0.014 a	
	CD4:CD8 ratio	F	11	9	26	26	84.4
		P	<0.001	<0.001 a	<0.001 b	<0.001 b	
Blood Activation Markers	CD4+CD183+ %	F	0.85	25	23	27	82.7
		P	0.429	<0.001 a	<0.001 a	<0.001 a	
	CD4+CCR4+ %	F	3	10	18	18	74.9
		P	0.071	<0.001 a	<0.001 b	<0.001 c	
	CD4+CCR6+ %	F	2	9	10	12	63.7
		P	0.160	<0.001 a	<0.001 a	<0.001 a	
Lymph Node Lineage Markers	CD4%	F	3	7	12	18	86.6
		P	0.067	<0.001 a	<0.001 b	<0.001 b	
	CD8%	F	11	35	12	9	84.2
		P	<0.001	<0.001 a	<0.001 b	<0.001 b	
	CD21%	F	0.8	7	4	15	66.2
		P	0.453	<0.001 a	<0.001 b	<0.001 c	
	B:T ratio	F	0.4	10	8	13	71.1
		P	0.672	<0.001 a	<0.001 ab	<0.001 b	
	CD4:CD8 ratio	F	2	8	9	17	76.8
		P	0.164	<0.001 a	<0.001 ab	<0.001 b	
Lymph Node Activa	CD4+CD183+ %	F	1	59	77	109	94.3
		P	0.314	<0.001 a	<0.001 b	<0.001 ab	

	CD4+CCR4+ %	F	1	10	7	8	41.9
		P	0.324	<0.001 a	0.010 b	0.007 b	
	CD4+CCR6+ %	F	0.6	21	56	23	74.7
		P	0.572	<0.001 a	<0.001 b	<0.001 a	
